# Construction and Analysis of the Cell Surface's Protein Network for Human Sperm-Egg Interaction

**DOI:** 10.1155/2013/962760

**Published:** 2013-08-12

**Authors:** Soudabeh Sabetian Fard Jahromi, Mohd Shahir Shamsir

**Affiliations:** Department of Biological and Health Sciences, Faculty of Bioscience & Medical Engineering, Universiti Teknologi Malaysia, 81310 Johor, Malaysia

## Abstract

Sperm-egg interaction is one of the most impressive processes in sexual reproduction, and understanding the molecular mechanism is crucial in solving problems in infertility and failed in vitro fertilization. The main purpose of this study is to map the sperm-egg interaction network between cell-surface proteins and perform an interaction analysis on this new network. We built the first protein interaction network of human sperm-egg binding and fusion proteins that consists of 84 protein nodes and 112 interactions. The gene ontology analysis identified a number of functional clusters that may be involved in the sperm-egg interaction. These include G-protein coupled receptor protein signaling pathway, cellular membrane fusion, and single fertilization. The PPI network showed a highly interconnected network and identified a set of candidate proteins: ADAM-ZP3, ZP3-CLGN, IZUMO1-CD9, and ADAM2-IZUMO1 that may have an important role in sperm-egg interaction. The result showed that the ADAM2 may mediate interaction between two essential factors CD9 and IZUMO1. The KEGG analysis showed 12 statistically significant pathways with 10 proteins associated with cancer, suggesting a common pathway between tumor fusion and sperm-egg fusion. We believe that the availability of this map will assist future researches in the fertilization mechanism and will also facilitate biological interpretation of sperm-egg interaction.

## 1. Introduction

Fertilization is the process in which sperm and egg recognize, bind, and fuse with each other. These interactions include the initial cell-cell adhesion followed by the membrane fusion between the two gametes [1]. During this process, many molecular interactions in the form of protein-protein interactions will mediate the sperm-egg binding process [2]. The acrosome is a large organelle in the sperm that secretes enzymes such as the serine protease and the acrosin which assist the sperm penetration into the extracellular matrix of the oocyte (the zona pellucida) [3]. Many prior researches have attempted to find the molecules that are involved in the binding and fusion process. For example, acrosin has been identified as an important factor in the binding process [4]. However, further research on acrosin-knockout mice showed that the interaction process can occur in absence of acrosin, suggesting that the interaction relationship between sperm and the egg is much more complex than previously thought [5]. Due to the various limitations of materials and difficulties in analyzing in vivo membrane protein-protein interactions (PPI), many efforts have failed to comprehensively elucidate the fusion mechanism, leaving the molecular interactions that mediate sperm-egg membrane fusion still poorly understood [6, 7]. This challenge is augmented further as protein-protein interactions can interact in many ways, ranging from direct physical associations among proteins in a complex to transient interactions that occur among members of certain protein pathways [8].

The molecular interactions that mediate sperm-egg membrane fusion are yet to be mapped as there is no complete PPI network between the sperm and the oocyte proteins [7, 9]. In this study, we constructed and analyzed a protein-protein interaction (PPI) network of all the membrane and surface proteins of the sperm and the oocyte proteins, identifying the essential PPI and their biological roles in the sperm-egg binding and fusion process. These analyses help us in better understanding of mechanism aspects of protein-protein interactions during the sperm-egg interaction process.

## 2. Methods

### 2.1. Selection of Proteins Expressed in Human Egg and Sperm

UniProt (http://www.uniprot.org/) was used to find human egg/oocyte and sperm related proteins with keywords "egg/oocyte" and "sperm." Subsequently the proteins that are expressed in human were selected. Each of the proteins UniProt ID was submitted to the STRING database (http://string-db.org/) and each protein-protein interaction was retrieved. The STRING database consists of known and predicted protein interactions that include direct (physical) and indirect (functional) associations. STRING quantitatively integrates interaction data from four different sources: genomic context, high-throughput experiments, coexpression, and prior knowledge from research publications [10–15].

### 2.2. Construction of the PPI Network

To create the sperm-egg interaction map, we compared the UniProt ID from sperm and egg maps to determine overlapping ID nodes between the sperm and the egg using the UltraCompare Professional 8.10 [16–18]. The overlapping ID nodes were then used to construct a sperm-egg PPI network in Cytoscape 2.8.2 [19]. By this method, we were able to consider all the specific possible interactions involved in the sperm-egg interaction.

### 2.3. Selection of the Proteins Associated with Cell Surfaces and Membrane Organization

The LOCATE database (http://locate.imb.uq.edu.au/) is a curated database that identifies and describes the membrane organization of proteins from mouse and human protein sequence sets. The UniProt IDs of the overlapping proteins in the sperm-egg PPI network were submitted to the LOCATE server to identify the proteins that are associated with cell surfaces and membrane organizations.

### 2.4. Clustering of PPI Networks

The final proteins datasets that have been filtered as associated with sperm-egg interaction and have been identified as involved in membrane organization were loaded into Cytoscape 2.8.2 and analysed using the AllegroMCODE plugin in Cytoscape. The AllegroMCODE plugin is a high performance fast algorithm that is used to detect clusters in a large protein network. Clusters in the network can be considered as protein complexes and functional modules, which can be identified as highly interconnected subgraphs [20]. The cluster analysis of the largest component in the network will enable the identification of important protein complexes and pathways.

### 2.5. Gene Ontology (GO) Enrichment Analysis

The clusters that have been identified using AllegroMCODE algorithm are then analyzed for their gene ontology using the BiNGO plugin in Cytoscape. The BiNGO plugin was used to identify the main ontologies in the protein network: molecular function, biological process, and cellular components [21].

### 2.6. DAVID (Database for Annotation, Visualization, and Integrated Discovery) v6.7 Analysis

DAVID v6.7 is a web-accessible program that provides a comprehensive set of functional annotation tools to understand the biological meaning behind large datasets of genes or proteins [22]. The identified UniProt IDs of proteins from the LOCATE database that are associated with cell surfaces and membrane of the overlapping network were submitted to DAVID to analyze the protein domains and pathways (Table 4).

## 3. Results and Discussion

Various researches have managed to identify the various molecules that are involved in sperm-egg binding and fusion. Most of these attempts have been focused on important factors in sperm-egg fusion in mammals, especially on the role of acrosin in the sperm penetration into the zona pellucida (ZP) [5, 6, 9, 23]. Many previous efforts have studied the PPI network to find novel interaction, important molecular function, essential biological process, and potential drug targets [19, 24–27]. Predicting molecular complexes from protein interaction data is important because it provides another level of functional annotation above other guilt-by-association methods. Since subunits of a molecular complex generally function towards the same biological goal, prediction of an unknown protein as part of a complex also allows increased confidence in the annotation of that protein [28].

### 3.1. Selection, Construction, and Clustering of the PPI Network

**Figure 1:**
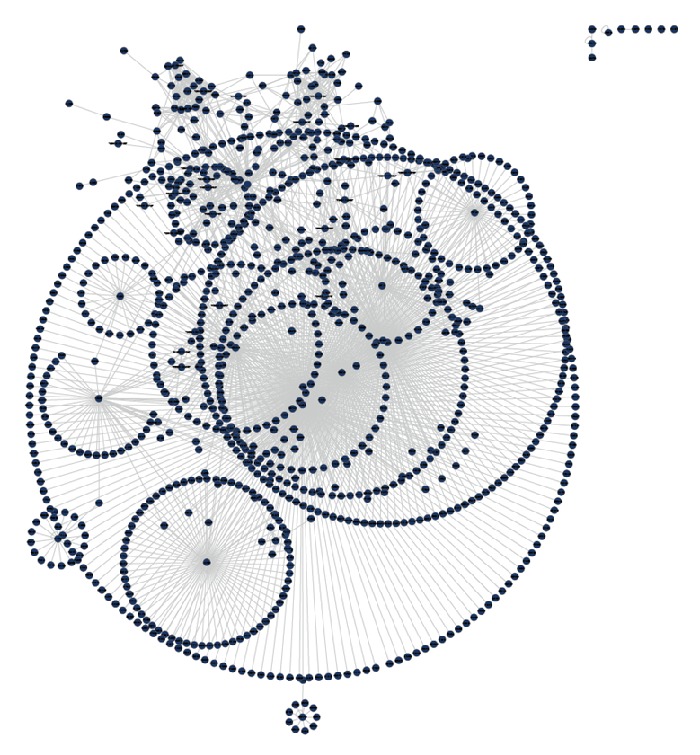
The PPI network of human egg related proteins in Cytoscape 2.8.2: this map involves 1056 proteins and 1700 interactions between these proteins.

**Figure 2:**
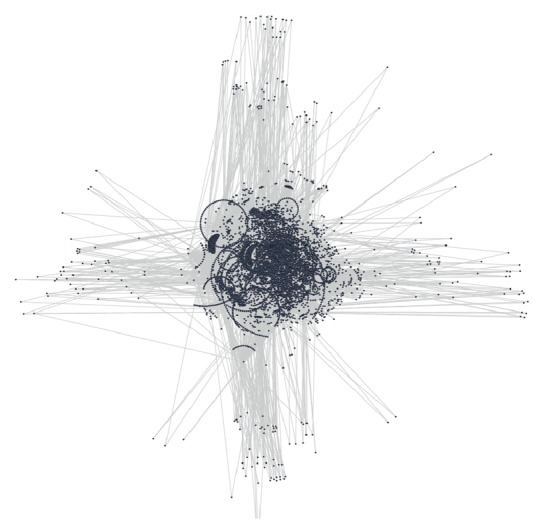
The PPI network of human sperm related proteins in Cytoscape 2.8.2: this map involves 6450 proteins and 34579 interactions between the proteins.

The initial selection of proteins that are associated with human sperm and egg from the UniProt database produced 1056 proteins for egg and 6450 for sperm. The PPI interaction for egg is represented in Figure 1 and for sperm in Figure 2. Overall, there are significantly more PPI related to the sperm than to the egg. Examination of the PPI data showed that there are 1700 interactions in egg and 34,579 interactions in sperm. 

**Figure 3:**
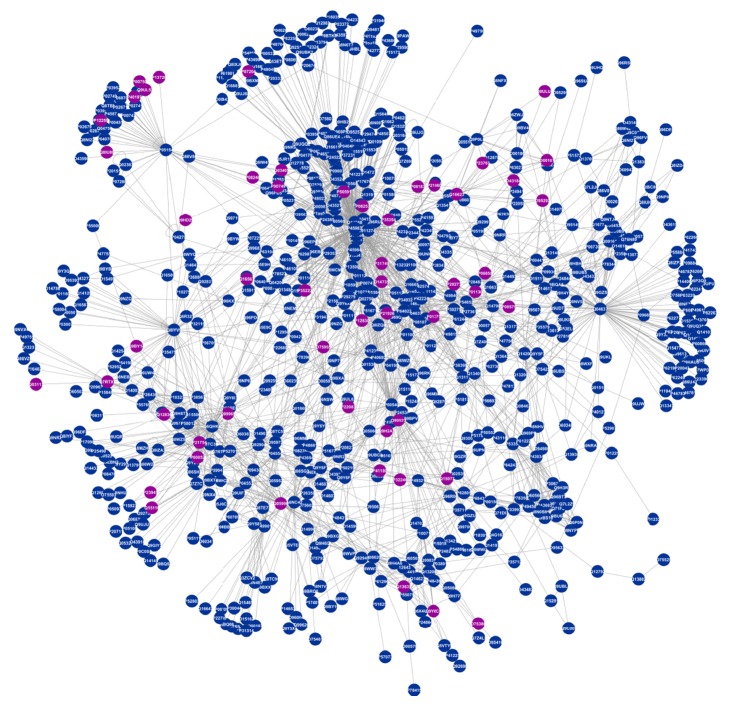
The PPI network of overlapping proteins rendered using Cytoscape 2.8.2 showed 725 proteins and 2173 interactions between them. The 84 protein nodes highlighted in purple are membrane and surface proteins identified using the LOCATE database.

Selection of the overlapping protein ID nodes between the sperm and the egg proteins using UltraCompare Professional 8.10 showed 725 overlapping proteins with 2173 interactions between them. Filtering the overlapping protein nodes using LOCATE database identified 84 protein nodes that are associated with the cell's surfaces and membranes (Figure 3).

Membrane organization analysis using LOCATE database showed that the 84 identified proteins are associated with the cell's surface and plasma membrane. 14 proteins were identified as soluble proteins that include 7 secreted proteins, and 63 proteins were membrane proteins including 20 type I, 21 type II, and 22 multipass membrane proteins (Table 1).

**Figure 4:**
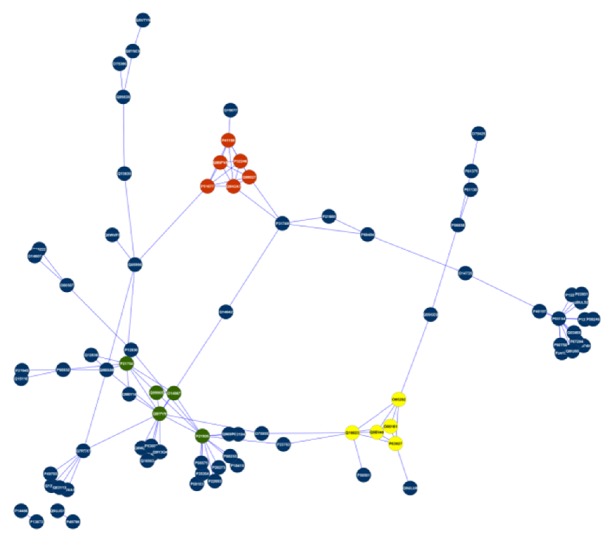
Sperm-egg interaction network was ranked as 1 to 3 clusters. Cluster 1, cluster 2, and cluster 3 are presented in red, yellow, and green, respectively.

The PPI network of the 84 protein nodes was extracted from the overlapping PPI network in Figure 3 and presented in Figure 4. This smaller PPI network of cell's surface and membrane proteins showed 112 interactions between them. The AllegroMCODE algorithm revealed three densely interconnected clusters that may represent molecular complexes within the PPI network that may participate in different aspects of sperm-egg interaction process (Figure 4) and are listed in Table 2. 

Using the BiNGO plugin for Cytoscape, we identified the most significant GO biological process within the membrane and surface PPI (Table 3). The result showed that the largest cluster contains proteins that participate in the G-protein coupled receptor protein signaling pathway (GO: 0007186). G-protein coupled receptors (GPCRs) are a group of seven transmembrane proteins which bind signal molecules outside the cell, transduce the signal into the cell, and trigger a cellular response. The GPCRs work with the help of a G-Protein which binds to the energy-rich GTP [29].

The proteins identified within the most interconnected cluster are the C-C chemokine receptor type 1 (CCR1) and type 3 (CCR3), G-protein coupled estrogen receptor 1 (GPER), and the extracellular calcium-sensing receptor (CASR) protein and are all involved in the G-protein coupled receptor protein signaling pathway for the sperm-egg interaction. Theoretically, the human spermatozoon contains the mRNA coding for RANTES (regulated on activation and normally T-cell expressed and secreted) receptors CCR1 and expresses the CCR3 protein. The progesterone-enhanced hamster egg penetration test (HEPT) has shown that RANTES has a role in the sperm/oocyte fusion [30]. It is involved in the sperm-egg contact by increasing the intracellular calcium by causing the breakdown of plasma membrane polyphosphoinositides (PPI), which subsequently affects the production of inositol triphosphate (IP3). IP3 causes the release of calcium from internal stores, as it has been identified to occur in various cell types [31]. The spermatozoa that are lured to the egg by species specific chemoattractants are first made hypermotile by bicarbonate-induced cAMP signaling. Then, it is activated by egg-coat extracellular matrix ZP3 (zona pellucida) oligosaccharides that bind human specific galactosyltransferase on the sperm surface. Membrane clustering of these enzymes activates the sperm sensor GPCRs, causing transmembrane influx of sodium ions. This is followed by tyrosine phosphorylation of the ZP3 receptors, leading in turn to sperm pronuclear binding of the ZP3 receptor in the inner membrane leaflet [32].

The most important biological process identified for cluster 2 is the cellular membrane fusion (0006944). The cellular membrane fusion is a cellular process that joins two lipid bilayers to form a single membrane, a critical step in fusing the sperm and the egg [33]. The proteins identified within the cluster are the vesicle-associated membrane protein 8 (VAMP8) and synaptosomal-associated protein 23 (SNAP23). These proteins have been shown in laboratory work as critical interacting proteins in membrane fusion and human sperm acrosome reaction [34]. 

**Figure 5:**
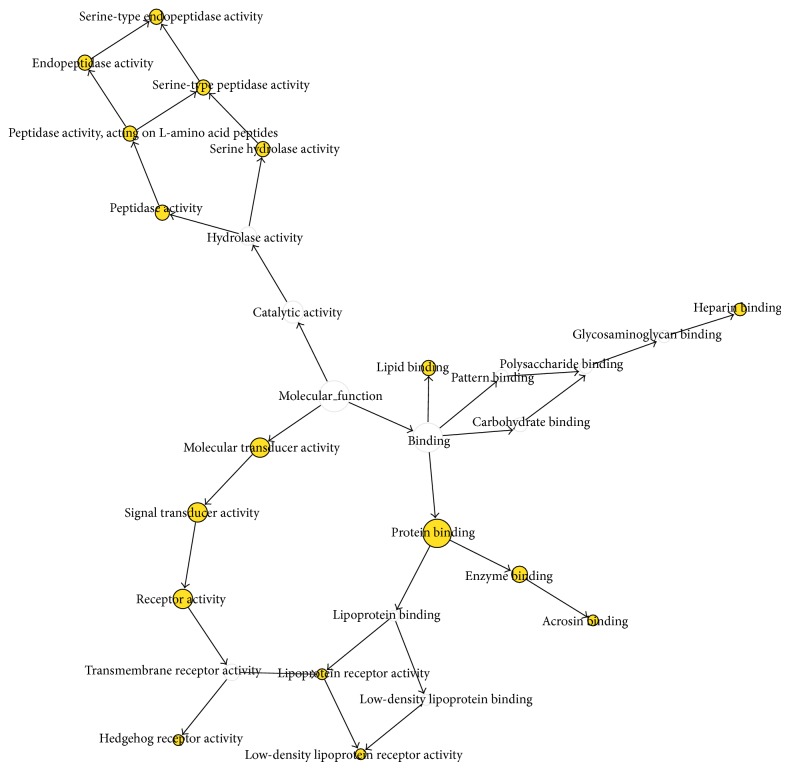
Molecular function map. Darker nodes refer to the significant ontologies of the dataset.

The most important biological process of cluster 3 is the single fertilization (0007338). The single fertilization process is defined as the union of male and female gametes to form a zygote [35, 36]. The proteins that are identified in the single fertilization PPI are the Izumo sperm-egg fusion protein 1 (IZUMO1), disintegrin and metalloproteinase domain-containing protein 2 (ADAM2), calmegin (CLGN), CD9 antigen (CD9), and zona pellucida sperm-binding protein 3 (ZP3). The IZUMO1 in the sperm is an immunoglobulin superfamily member (with an immunoglobulin-like domain (Ig)) that is essential for sperm-oocyte fusion. CD9 may work by interacting with a sperm protein in *trans* (although data supporting this mode of action are minimal), by regulating other egg membrane proteins in *cis*, and/or through exosome-mediated release. ADAM2 has been implicated in sperm-egg binding and fusion [37]. Previous studies have demonstrated that calmegin −/− sperms were defective in migrating into the oviducts and in binding to the egg plasma membrane [38]. As for the sperm-zona binding, the widely accepted involvement of sugar moiety on zona pellucida 3 (ZP3) is indicated to be dispensable by gene disruption experiments. In the sperm-egg fusion process, CD9 on egg and IZUMO1 on sperm have been identified as essential factors [5]. The overall analysis showed that 61 proteins (72.6%) are associated with functions involving binding activity, acrosin binding, and serine-type peptidase and serine hydrolase activities that are all critical for sperm-egg interaction [6] (Figure 5). 

### 3.2. Predicted Interactions and Candidate Proteins

The PPI in the three clusters may be important for the sperm-egg fusion (Figure 4). Some of these interactions have not been reported in previous experimental work and were inferred using prediction text mining, interologs mapping, and unspecific method coexpression. We identified five significant PPIs within the three clusters.

The PPI of P2Y13 in cluster 1 may be significant for sperm-egg interaction. The P2Y13 has been predicted to interact with CCR1, CCR3, CASR, CXL16, and GPER. However, this protein is yet to be detected in the ovary. It would be interesting to examine the role of P2Y13 in sperm-egg interaction. The second PPI is between ADAM2 and ZP3. The ADAM2 is found in sperm while the ZP3 is found in the egg. Previous studies using ADAM knockouts have shown reduced migration of sperm into the oviduct through the uterotubal junction, reduced binding to the ZP, and/or reduced binding and fusion to the egg plasma membrane [7]. The third PPI is between ZP3 and calmegin (CLGN). The calmegin −/− sperm has been shown to be defective when migrating into the oviducts and in binding to the egg plasma membrane. The fourth PPI is the interaction between IZUMO1 and CD9. IZUMO1 is essential for the sperm to bind to eggs and that CD9 is essential for eggs to bind to sperm; therefore, it is tempting to speculate that they interact with each other to form a fusogenic complex. If these proteins do indeed interact, it is likely that they both require associating proteins on the sperm and egg cell surfaces, and the identity of these putative factors should be investigated [39]. The fifth PPI is between ADAM2 and IZUMO1. Chen and colleagues reported that the *CD9* is a tetraspan protein that is associated with several beta1 integrins, including alpha6beta1. Alpha6beta1 is present in eggs and interacts with the sperm-surface *glycoprotein* ADAM2 (fertilin beta) [40]. This result shows that ADAM2 may act as an interconnecting protein that mediates interaction between CD9 and IZUMO1.

For an overall overview of the interaction proteins, the functional analysis including domain analysis and pathway was performed using the tools in DAVID. The domain analysis using DAVID functional annotation tools included three subset databases: InterPro, PIR_Superfamily, and SMART databases, which showed 24, 4, and 6 statistically significant domains, respectively. These are the EGF-like region conserved sites (IPR013032, containing 7 proteins) that are involved in disulphide bonds, sperm binding glycoprotein ZP3-alpha domain (PIRSF002554, containing 2 proteins) involved in sperm-egg binding, and _Tryp_SPc domain (SMART:SM00020, containing 7 proteins) involved in the proteolysis and serine-type endopeptidase activity. These results showed some significant enriched domains that in the protein networks would regulate some aspects of interaction between sperm and egg. The functional significance of EGF domains in what appear to be unrelated proteins is not yet clear [41]. However, a common feature is that the repeats are found in the extracellular domain of membrane bound proteins or in proteins known to be secreted [42]. Evans et al. (1998) suggested a potential role of the cysteine-rich and/or EGF-like domains in sperm-egg adhesion [43], and the fusion peptide of the fertilin *α* protein was previously identified within the cysteine-rich domain [44]. In order to digest the zona pellucida glycoproteins, the acrosome organelle in the apical region of sperm head recognizes and binds with a single O-linked oligosaccharides chain, which is probably ZP3, and secretes enzymes such as serine protease and acrosin which help sperm penetration [45]. The KEGG analysis indicated that 12 statistically significant pathways were presented in sperm-egg PPI network. Interestingly, 10 proteins of the sperm-egg PPI network involved in sperm-egg interaction process were involved in the pathway in cancer [46]. These proteins are involved in prostate cancer, endometrial cancer, and small cell lung cancer. This suggests that fusion process in tumor growth and sperm-egg may share similar interactions.

## 4. Conclusion

We have created the first protein interaction network of human membrane and surface sperm-egg interaction proteins by using computational approach. The PPI network of sperm and egg showed the PPI map and revealed highly interconnected proteins, ranging from direct physical associations among proteins in a complex to transient interactions that occur among members of certain protein pathways. The analysis of the protein network enabled us to identify a set of candidate proteins important for the interaction between sperm and egg in human. We reported some predicted protein-protein interactions like ADAM-ZP3, ZP3-CLGN, IZUMO1-CD9, and ADAM2-IZUMO1 that may play an important role in sperm-egg interaction. These predicted interactions shows that ADAM2 may mediate interaction between two essential factors CD9 and IZUMO1. The association of P2Y13 in sperm with a protein on plasma membrane of egg is entirely speculative. 

KEGG analysis indicated that 12 statistically significant pathways were presented in sperm-egg PPI network. Interestingly, 10 proteins of the sperm-egg PPI network involved in sperm-egg interaction process were involved in the pathway in cancer, suggesting a common pathway between tumor fusion and sperm-egg fusion. The availability of this map will assist future researches studying the fertilization mechanism and will also facilitate biological interpretation of sperm-egg interaction.

### 4.1. Availability and Requirement

The sperm-egg PPI is available upon request. Contact: shahir@utm.my.

## Figures and Tables

**Table 1:** Membrane organization analysis of interacting proteins using LOCATE database.

Protein class	Names of proteins
Soluble, nonsecreted protein (14)	CAPS1, GOPC, UQCC, FLOT1, TULP3, I5P1, RGS4, KGP2, SNP23, GNA12, IPSP, CTNB1, PTEN, AKT1

Secreted protein (7)	UROK, FA11, LIPL, MMP2, FA5, PRTN3, UPAR

Type I membrane protein (20)	ADA12, CADH1, CD22, CLGN, CXL16, ELNE, ERBB3, GPV, LDLR, PGH2, PTPRC, TF, TM190, TMEDA, TPA, TRBM, ZP2, ZP3, ZP4, IZUMO1

Type II membrane protein (21)	BCL2, COX41, CX7A2, FUT4, IGSF8, PDCD1, PPM1L, PTC1, STX1A, TM11E, TMPS6, TNF10, TNFA, USP9Y, UTY, VAMP1, VAMP2, VAMP8, VAPB, ZP1, ZPBP1

Multipass membrane protein (22)	ADAM2, CASR, CCR1, CD9, CDIPT, CTSR4, FSHR, GPER, HCN4, HHAT, LMBR1, MDR1, MSPD1, MSPD3, P2RY6, P2Y13, PTC2, SMO, SYPL1, TX101, TXTP, CCR3

**Table 2:** The names of proteins and their respective classes in the three clusters.

Clusters	Names of proteins	Protein class
Cluster 1 (red in Figure 4)	CXL16	Type I membrane protein
	CASR, CCR1, GPER, P2Y13, CCR3	Multipass membrane protein

Cluster 2 (yellow in Figure 4)	SNP23	Soluble, nonsecreted protein
	STX1A, VAMP2, VAMP8, VAPB	Type II membrane protein

Cluster 3 (green in Figure 4)	CLGN, ZP3, IZUMO1	Type I membrane protein
	ADAM2, CD9	Multipass membrane protein

**Table 3:** The overrepresented GO biological process of each cluster.

AllegroMCODE cluster ID	Description	Overrepresented GO code	*P* value
1 (red in Figure 4)	G-protein coupled receptor protein signaling pathway	0007186	1.6438*E* − 4
2 (yellow in Figure 4)	Cellular membrane fusion	0006944	7.7391*E* − 5
3 (green in Figure 4)	Single fertilization	0007338	1.2968*E* − 12

**Table 4:** Enriched DAVID pathways associated with sperm-egg interaction network.

Pathways (*P* ≤ 0.05)	Count	%	*P* value
hsa04610: complement and coagulation cascades	8	9.52	2.94*E* − 06
hsa04130: SNARE interactions in vesicular transport	5	5.95	4.08*E* − 04
hsa05200: pathways in cancer	10	11.90	0.003
hsa05213: endometrial cancer	4	4.76	0.012
has05217: basal cell carcinoma	4	4.76	0.014
has05222: small cell lung cancer	4	4.76	0.044
has04640: hematopoietic cell lineage	4	4.76	0.046
has04210: apoptosis	4	4.76	0.048
has05215: prostate cancer	4	4.76	0.050
